# Mesh *versus* suture in elective repair of umbilical hernia: systematic review and meta‐analysis

**DOI:** 10.1002/bjs5.50276

**Published:** 2020-04-06

**Authors:** L. J. Madsen, E. Oma, L. N. Jorgensen, K. K. Jensen

**Affiliations:** ^1^ Digestive Disease Centre, Research Department, Bispebjerg and Frederiksberg Hospital, Faculty of Health and Medical Sciences University of Copenhagen, Nielsine Nielsens Vej 11, Entrance 8, Ground Floor DK‐2400 Copenhagen NV Denmark

## Abstract

**Background:**

Mesh repair of umbilical hernia has been associated with a reduced recurrence rate compared with suture closure, but potentially at the expense of increased postoperative complications and chronic pain. The objective of this systematic review and meta‐analysis was to examine the outcomes after elective open mesh and suture repair for umbilical hernia in adults.

**Methods:**

A literature search was conducted to identify studies presenting original data on elective open mesh and suture repair of umbilical hernia. The primary outcome was hernia recurrence. Secondary outcomes included surgical‐site infection (SSI), seroma, haematoma and chronic pain. Meta‐analyses were undertaken.

**Results:**

The search resulted in 5353 hits and led to 14 studies being included (6 RCTs and 8 observational studies) describing a total of 2361 patients. Compared with suture, mesh repair was associated with a lower risk of recurrence (risk ratio (RR) 0·48, 95 per cent c.i. 0·30 to 0·77), with number needed to treat 19 **(**95 per cent c.i. 14 to 31). Mesh repair was associated with a higher risk of seroma (RR 2·37, 1·45 to 3·87), with number needed to harm 30 (17 to 86). There was no significant difference in the risk of SSI, haematoma or chronic pain.

**Conclusion:**

The use of mesh in elective repair of umbilical hernia reduced the risk of recurrence compared with suture closure without altering the risk of chronic pain.

## Graphical abstract

Mesh reduced the risk of recurrence compared with suture closure in elective repair of umbilical hernia, with no difference in the risk of chronic pain.

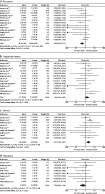

Mesh repair justified

## Abstract (es)

**Antecedentes:**

La reparación con malla de la hernia umbilical se ha asociado con una tasa menor de recidivas en comparación con el cierre con suturas, pero potencialmente a expensas de un aumento de complicaciones postoperatorias y dolor crónico. El objetivo de esta revisión sistemática y metaanálisis fue examinar los resultados después de reparación abierta electiva con malla o suturas para la reparación de una hernia umbilical en adultos.

**Métodos:**

Se llevó a cabo una búsqueda en la literatura para identificar estudios que presentaban datos originales sobre la reparación abierta electiva con malla y sutura de la hernia umbilical. El resultado primario fue la recidiva herniaria. Los resultados secundarios incluyeron la infección del sitio quirúrgico (*surgical site infection*, SSI), seroma, hematoma y dolor crónico. Se realizaron metaanálisis.

**Resultados:**

En la búsqueda identificaron 5.353 documentos, incluyéndose 14 estudios (6 ensayos clínicos aleatorizados, 8 estudios observacionales) que presentaban datos de un total de 2.361 pacientes. En comparación con la sutura, la reparación con malla se asoció con un menor riesgo de recidiva (tasa de riesgo, *risk ratio*, RR 0,48, i.c. del 95% 0,30 a 0,77) y número necesario para tratar de 19 (i.c. del 95% 14 a 31). La reparación con malla se asoció con un mayor riesgo de seroma (RR 2,37, i.c. del 95% 1,45 a 3,87) y número necesario para provocar daño de 30 (i.c. del 95% 17 a 86). No hubo diferencia significativa en el riesgo de SSI, hematoma o dolor crónico.

**Conclusión:**

El uso de malla en la reparación electiva de la hernia umbilical redujo el riesgo de recidiva en comparación con el cierre con sutura, sin modificar el riesgo de dolor crónico.

## Introduction

Repair of umbilical hernia is the second most frequent hernia operation in the Western world, exceeded only by groin hernia repair^1^. Suture repair has been challenged by a growing volume of evidence supporting the use of mesh, as mesh has been associated with reduced recurrence rates compared with sutures alone^2^, ^3^. Suture repair is used widely for umbilical hernia defects smaller than 2 cm, but when the defects are larger than 4 cm this technique has been associated with recurrence rates of up to 54 per cent^4^. In a Danish cohort^2^, 69 per cent of 989 patients with a primary umbilical hernia had elective suture repair over a 3‐year period. The study found significantly decreased recurrence rates following mesh *versus* suture repair in ventral hernias of up to 1 cm and in those of more than 1 to 2 cm.

The relative complexity and prolonged duration of surgery associated with the use of mesh for repair of smaller umbilical hernias may explain this discrepancy between research findings and surgeons' choice of repair. It has also been suggested^2^, ^3^, ^5^ that mesh is related to an increased risk of complications, including surgical‐site infection (SSI), seroma, haematoma and chronic pain. It is unclear whether these complications could offset the benefits of mesh repair compared with sutures alone.

A review^6^ that included both RCTs and observational studies, published in 2014, looked at elective and emergency repair of primary epigastric and umbilical hernias. Since then, several observational studies and RCTs concerning umbilical hernia have been reported. The objective of the present study was to compare five outcomes following open mesh and suture repair of umbilical hernia in adults. The primary outcome was hernia recurrence; secondary outcomes were SSI, seroma, haematoma and chronic pain.

## Methods

This systematic review and meta‐analysis was conducted in accordance with the PRISMA statement^7^.

### Eligibility criteria

Original studies reporting recurrence rate and additional outcomes after elective primary (non‐recurrent) umbilical hernia repair by mesh or suture in adults were eligible for inclusion. From studies reporting additional repair methods other than open mesh or suture repair, for example laparoscopic repair, only data regarding open mesh and suture repair were extracted. Studies reporting on the treatment of other hernias, such as epigastric hernia, were included only if data regarding umbilical hernia were distinguishable from those relating to other hernia types. Paraumbilical hernia was defined as umbilical hernia^8^.

To reduce heterogeneity, studies on patients with cirrhosis, those undergoing concomitant surgery at the time of hernia repair, emergency repair, surgery in contaminated fields, non‐comparative studies, or those involving recurrent hernias were excluded. Studies published in a language other than English, those with no available full‐text article, and those reporting decision on repair technique based on a threshold defect size were also excluded.

### Search strategy

A literature search was conducted of MEDLINE (PubMed), Embase, the Cochrane Library, Web of Science and the Cumulative Index to Nursing and Allied Health Literature (CINAHL) from the date of establishment of the databases to 6 August 2019. Search terms used were ((hernia, ventral[MeSH Terms]) OR (umbilical hernia) OR (primary ventral hernia) OR (epigastric hernia)) AND ((mesh) OR (suture)). One author screened titles and abstracts. Two authors reviewed full‐text articles independently.

### Data extraction

The primary outcome was hernia recurrence. Secondary outcomes were SSI, seroma, haematoma and chronic pain. Information extracted from each study included year of publication, type of study, number of patients, type of hernia, sex, age, BMI, diabetes mellitus, ASA grade, hernia defect size, technical information, duration of surgery, and length and type of follow‐up.

### Assessment of bias

Two authors evaluated the risk of bias in the included studies independently. In each cohort study, risk of bias was assessed by using the Newcastle–Ottawa quality assessment scale (NOS)^9^. The score, from 0 (lowest) to 9 (highest), was based on the quality of information accessible on three broad categories: selection, comparability and outcomes.

The Cochrane Collaboration tool for assessment of the risk of bias^10^ was applied to the RCTs. Each study was assessed for low, high or unclear risk of bias in five categories: selection, performance, detection, attrition and reporting bias.

### Statistical analysis

Where studies reported outcomes after more than one type of open mesh or suture technique, data were pooled into one mesh and one suture group respectively. Meta‐analyses were performed on pooled and separated data from RCTs and cohort studies respectively. Heterogeneity across study results was estimated by the Cochrane approach (*I* ^2^) and interpreted in accordance with the Cochrane Handbook^10^. The results of meta‐analyses are presented as forest plots, including overall risk ratios (RRs) with confidence intervals on hernia recurrence, SSI, seroma and haematoma. *I* ^2^ estimates are presented in each forest plot. Estimation of the number needed to treat (NNT) or number needed to harm (NNH) was performed by taking the inverse of the absolute risk reduction formula^11^. Results for chronic pain were compared between the repair groups. No statistical test was applied for chronic pain data, as these were too heterogeneous across the studies. Statistical software used was Review Manager version 5.3 (The Cochrane Collaboration, The Nordic Cochrane Centre, Copenhagen, Denmark).

## Results

**Figure 1:**
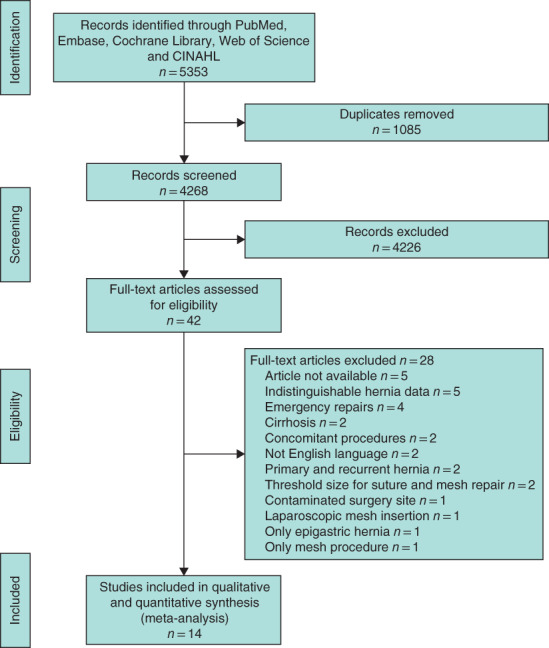
**PRISMA diagram for the review**

The literature search yielded 5353 unique hits (*Fig*. 1). After initial screening of titles and abstracts, 42 studies were assessed for inclusion eligibility. In total, 14 studies^1^, ^3^, ^5^, ^12^, ^13^, ^14^, ^15^, ^16^, ^17^, ^18^, ^19^, ^20^, ^21^, ^22^ were included, comprising six RCTs, one prospective cohort study and seven retrospective cohort studies, involving a total of 2361 patients. Characteristics of the included studies are shown in *Table* 1. Two studies^1^, ^3^ explicitly defined umbilical hernia according to European Hernia Society definitions^8^.

**Table 1:** Characteristics of studies included in the meta‐analysis

					Outcome variable reported				
Reference	NOS score	Type of study	Mesh	Suture	Recurrence	SSI	Seroma	Haematoma	Pain	Follow‐up (years)*
Arroyo *et al*.^15^ (2001)		RCT	100	100	Yes	Yes	Yes	Yes	No	5·3 (1·8–6·7)†
Asolati *et al*.^22^ (2006)	4	Cohort	132	97	Yes	Yes	Yes	Yes	No	0·5§
Berger *et al*.^12^ (2014)	7	Cohort	126	266	Yes	Yes	Yes	No	No	5 (0·1–11·9)
Dalenbäck *et al*.^18^ (2013)	6	Cohort	21	111	Yes	Yes	No	No	Yes	5·8 (2·3–11·8)†
Farrow *et al*.^20^ (2008)	8	Cohort	65	87	Yes	Yes	No	No	No	1·7 (0·1–4·8)†
Halm *et al*.^19^ (2005)	7	Cohort	12	98	Yes	Yes	No	No	Yes	2·7 (0·8–5·6)
Kaufmann *et al*.^3^ (2018)		RCT	146	138	Yes	Yes	Yes	Yes	Yes	2·1 (0–7·3)
Lal *et al*.^16^ (2012)		RCT	32	30	Yes	Yes	Yes	Yes	Yes	n.a.
Polat *et al*.^17^ (2005)		RCT	32	18	Yes	Yes	Yes	Yes	Yes	1·8 (0·5–3·7)†
Sadiq and Khurshid^21^ (2013)		RCT	30	30	Yes	Yes	Yes	Yes	No	1·0§
Sanjay *et al*.^13^ (2005)	6	Cohort	39	61	Yes	Yes	Yes	Yes	No	4·5 (1–8)
Tunio^14^ (2017)		RCT	43	43	Yes	Yes	Yes	Yes	Yes	3·0§
Venclauskas *et al*.^5^ (2017)	7	Cohort	52	146	Yes	Yes	Yes	Yes	Yes	4·7 (1·8–12·6)
Winsnes *et al*.^1^ (2016)	7	Cohort	184	122	Yes	Yes	No	No	No	6·8 (0·9–9·7)

* Values are median (range) unless indicated otherwise;

† values are mean (range);

§ values are maximum follow‐up. NOS, Newcastle–Ottawa scale; SSI, surgical‐site infection; n.a., not available.

### Study characteristics

Demographic and clinical data are shown in *Table* 2. One cohort study^12^ found that patients undergoing mesh repair were more likely to have a larger hernia defect (mean(s.d.) 4·7(0·3) cm *versus* 2·0(0·2) cm in those undergoing sutured repair; *P* < 0·010) and higher BMI (mean(s.d.) 32·5(0·4) *versus* 30·5(0·3) kg/m^2^ respectively; *P* < 0·010).

**Table 2:** Demographic and clinical data

		BMI (kg/m^2^)		% of men		Diabetes mellitus (%)		Age (years)		ASA grade III–IV (%)	
Reference	Fascial defect size (cm)	Suture	Mesh	Suture	Mesh	Suture	Mesh	Suture	Mesh	Suture	Mesh
Arroyo *et al*.^15^	> 0	n.a.		40·0	42·0	n.a.		56·0 (14–79)‡	57·0 (14–79)‡	15·0	12·0
Asolati *et al*.^22^	≥ 1	n.a.		96·1		n.a.		56 (30–85)‡		n.a.	
Berger *et al*.^12^	0–4	30·5 (0·3)*	32·5 (0·4)*	96·7¶	100¶	7·9¶	20·6¶	56·6 (1·1)*, ¶	57·1 (0·9)*, ¶	63·3¶	65·6¶
Dalenbäck *et al*.^18^	1–4	n.a.		64·8		n.a.		49·0*		n.a.	
Farrow *et al*.^20^	3·1–28 cm^2^	32·1 (20–49)‡		98·0		n.a.		55·2 (26–84)‡		n.a.	
Halm *et al*.^19^	n.a.	27**		66·4		n.a.		56·7 (21–85)‡		n.a.	
Kaufmann *et al*.^3^	1–4	28 (19–44)†	28 (19–59)†	81·9	83·6	9·4	8·9	52·0 (20–74)†	55·0 (25–77)†	2·9	5·5
Lal *et al*.^16^	4–7	n.a.		8		n.a.		(25–70)		n.a.	
Polat *et al*.^17^	0–4	n.a.		26		n.a.		49·7 (27–82)‡	53·7 (33–72)‡, §	11	19
Sadiq and Khurshid^21^	> 3	n.a. (> 30 excluded)		7	10	n.a.		(30–45)	(30–50)	n.a.	
Sanjay *et al*.^13^	0–5	31·2 (23–45)†	33·3 (24–59)†	68·0		n.a.		53·0 (19–90)†	54·0 (30–81)†	13	8
Tunio^14^	> 3	n.a.		21		0		45·5 (21–70)†		n.a.	
Venclauskas *et al*.^5^	0·3–9	30·4 (7·0)*	36·0 (6·9)*	33·6	46	5·5	8	54·5 (16·8)*	54·9 (12·7)*	n.a.	
Winsnes *et al*.^1^	1–2	26 (20–36)†	29 (21–39)†	65·6	70·1	8·2	13·0	48·0 (18–84)†	50·0 (20–88)†	4·9	11·4

Values are

* mean(s.d.),

† median (range) or

‡ mean (range);

§ mean for onlay mesh group.

¶ Only demographic and clinical data based on case‐matching were available.

** Only average data were available. n.a., Not available.

Technical elements and duration of surgery are shown in *Table* 3. Techniques varied considerably across the included studies. In two studies^13^, ^14^ a single surgeon performed all the procedures. One study^3^ invited surgeons to specific pretrial training sessions. A longer duration of surgery for mesh compared with suture repair was reported by five studies^3^, ^5^, ^15^, ^16^, ^17^, and four^12^, ^13^, ^15^, ^17^ reported administration of preoperative antibiotics in all patients.

**Table 3:** Intraoperative data

	Suture			Mesh		
Reference	Technique	Material	Duration of surgery (min)	Technique¶	Material¶	Duration of surgery (min)
Arroyo *et al*.^15^	Interrupted	Non‐absorbable polyester	38·0*	Preperitoneal placement Defect ≤ 3 cm: plug Defect > 3 cm: flat sheet	Polypropylene, fixed with nylon 0 sutures	45·0*
Asolati *et al*.^22^	–	–	–	Onlay, inlay or combined	Poliglecaprone 25 and polypropylene filament (combined)	–
Berger *et al*.^12^ ¶	Interrupted transverse closure	Non‐absorbable	–	Underlay (preperitoneal), 3‐cm overlap	Polypropylene, fixed with permanent sutures	–
Dalenbäck *et al*.^18^	Mayo repair or single or double, interrupted or continuous	Non‐absorbable monofilament	–	Onlay, plug, intraperitoneal or combined	Polypropylene or expanded polytetrafluoroethylene	–
Farrow *et al*.^20^	–	–	–	–	94% polypropylene; 5% polytetrafluoroethylene	–
Halm *et al*.^19^	–	–	–	Preperitoneal	–	–
Kaufmann *et al*.^3^	Interrupted or continuous transverse closure	Polypropylene 0/0	33·0 (10–95)†	Preperitoneal	Polypropylene, fixed with individual monofilament sutures	44·0 (20–122)†
Lal *et al*.^16^	Interrupted vertical closure	Polypropylene	(45–85)	Onlay	Polypropylene	(45–85)
Polat *et al*.^17^	Mayo repair	–	34·4 (20–50)‡	Combined (PHS) or onlay mesh	Polypropylene	40·5 (20–60)‡, **
Sadiq and Khurshid^21^	Mayo repair or interrupted	Polypropylene	–	Onlay	–	–
Sanjay *et al*.^13^	Mayo repair or interrupted	–	–	Flat mesh or plug	Polypropylene	–
Tunio^14^	Mayo repair	–	–	Onlay	Polypropylene, fixed with interrupted sutures	–
Venclauskas *et al*.^5^	Keel	Slowly absorbable monofilament	68·6(34·1)*	Onlay or sublay	Polypropylene	107·9(55·7)*
Winsnes *et al*.^1^	Interrupted or shoelace	–	–	Sublay, onlay, intraperitoneal or plug	Polypropylene	–

Values are

* mean(s.d.),

† median (range) or

‡ mean (range).

§ Onlay mesh group;

¶ includes only mesh used in the open approach;

** combined (PHS), Prolene Hernia System.

Follow‐up was assessed by clinical examination in all RCTs, and by the last clinical examination noted in medical records in all cohort studies. In some studies, additional follow‐up was accomplished by questionnaires sent to patients to identify recurrence^13^, ^18^, long‐term infection^13^ and pain^18^, or by telephone contact to identify chronic pain^5^. In three cohort studies^1^, ^5^, ^19^, patients received an invitation for an extra physical examination. Evidence of recurrence was based on physical examination, imaging (ultrasonography or CT) or reoperation^1^, ^3^, ^5^, ^12^, ^20^.

### Bias

**Figure 2:**
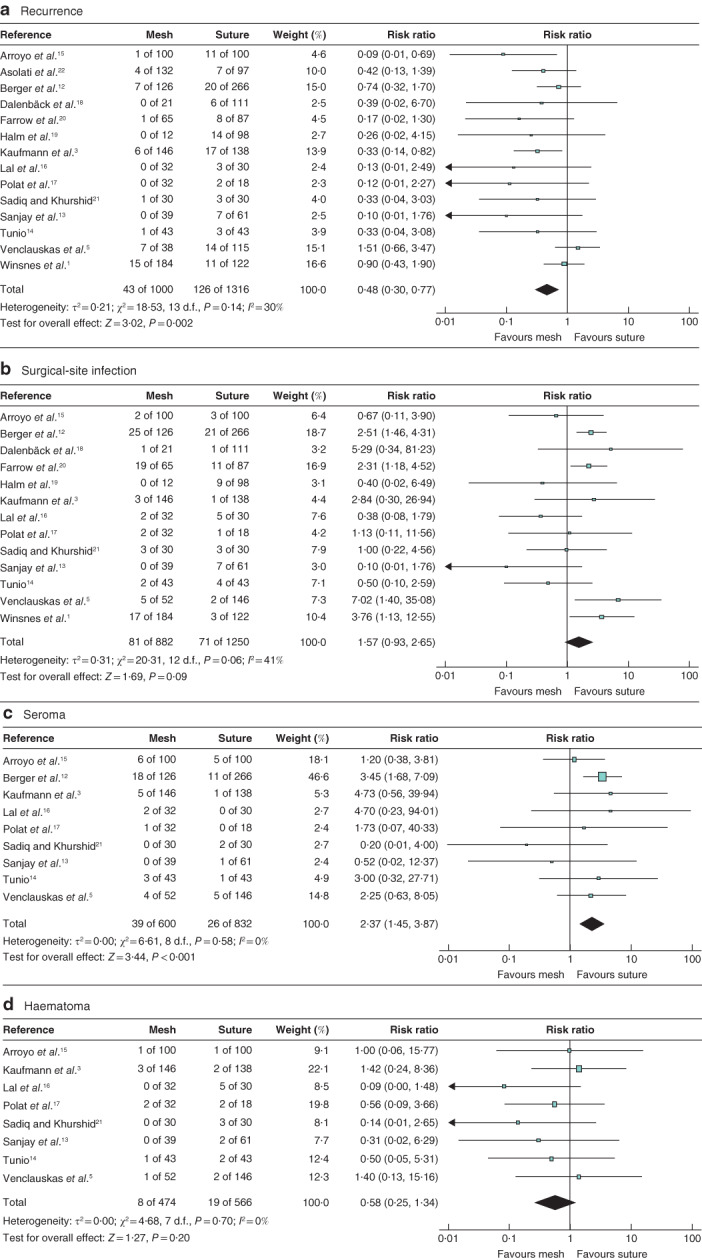
Forest plots comparing pooled data for recurrence, surgical‐site infection, seroma and haematoma after mesh *versus* suture repair of umbilical hernia **a** Recurrence, **b** surgical‐site infection, **c** seroma and **d** haematoma. A Mantel–Haenszel random‐effects model was used for meta‐analysis. Risk ratios are shown with 95 per cent confidence intervals.

NOS scores for the cohort studies are shown in *Table* 1. Only a single RCT^3^ was considered to exhibit a low risk of bias. The other RCTs^14^, ^15^, ^16^, ^17^, ^21^ were assessed as having an unclear to high risk of bias, as no descriptions of randomization procedures, blinding or loss to follow‐up were accessible. According to the Cochrane interpretation of the *I* ^2^ estimate^10^, heterogeneity across results for recurrence and SSI from the pooled data analyses was considerable to moderate, whereas heterogeneity across results for seroma and haematoma was low (*Fig*. 2).

**Figure 3:**
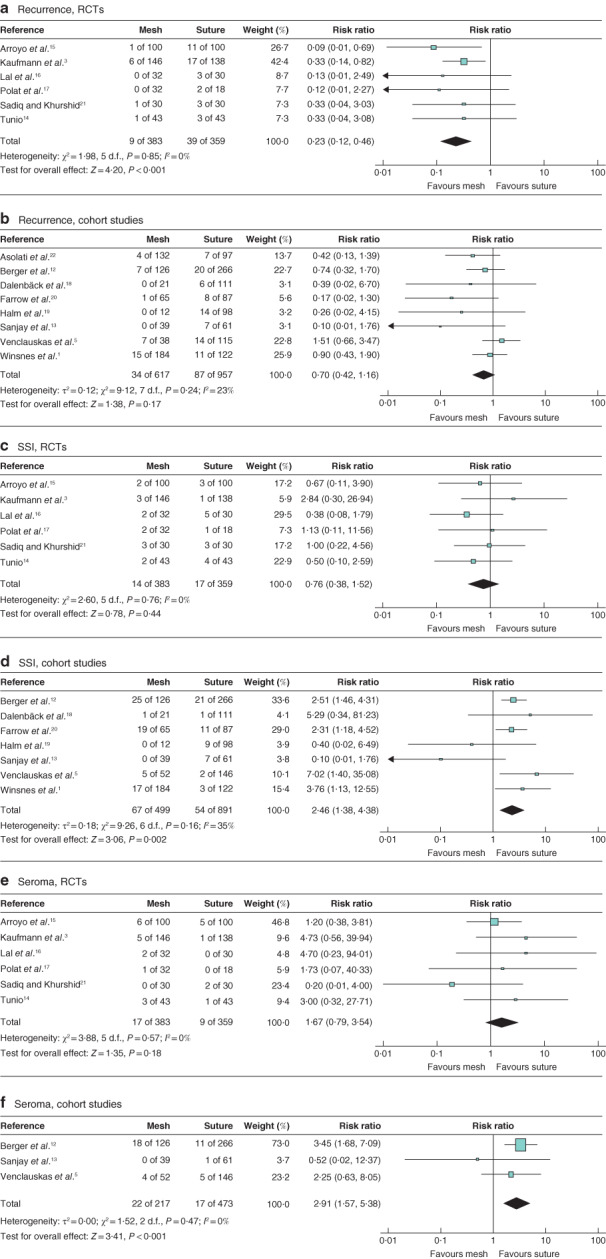
Forest plots comparing outcomes after mesh *versus* suture repair of umbilical hernia separately for RCT and cohort studies: sensitivity meta‐analysis **a** Recurrence, **c** surgical‐site infection (SSI) and **e** seroma in RCTs; **b** recurrence, **d** SSI and **f** seroma in cohort studies. Mantel–Haenszel fixed‐effect (**a,c,e**) and random‐effects (**b,d,f**) models were used for meta‐analysis. Risk ratios are shown with 95 per cent confidence intervals.

### Hernia recurrence, surgical‐site infection and seroma formation

#### Pooled data analyses

Mesh repair was associated with a significantly decreased risk of hernia recurrence compared with suture repair (RR 0·48, 95 per cent c.i. 0·30 to 0·77; *P* = 0·002) (*Fig*. 2). The NNT was 19 (95 per cent c.i. 14 to 31). Mesh repair was associated with a higher risk of seroma formation (RR 2·37, 1·45 to 3·87; *P* < 0·001), and the NNH was 30 (17 to 86). No significant difference was found in the risk of SSI (RR 1·57, 0·93 to 2·65; *P* = 0·090) or postoperative haematoma (RR 0·58, 0·25 to 1·34; *P* = 0·200).

#### Sensitivity analyses

A significant difference in the risk of recurrence was detected among the RCTs (RR 0·23, 95 per cent c.i. 0·12 to 0·46; *P* < 0·001), in contrast to the cohort studies (RR 0·70, 0·42 to 1·16; *P* = 0·170) (*Fig*. 3). The risk of SSI was significantly increased following mesh repair based on the cohort study data (RR 2·46, 1·38 to 4·38; *P* = 0·002), whereas no significant difference was detected among the RCTs (RR 0·76, 0·38 to 1·52; *P* = 0·440). Likewise, the risk of seroma formation was higher after mesh than suture repair in the cohort studies (RR 2·91, 1·57 to 5·38; *P* < 0·001), although not among the RCTs (RR 1·67, 0·79 to 3·54; *P* = 0·180).

### Pain

Pain was reported in seven of the included studies^3^, ^5^, ^14^, ^16^, ^17^, ^18^, ^19^ and assessed in a variety of ways (*Table* 1). Definitions of chronic pain also varied. Three reports were excluded from the comparison of chronic pain results, owing to follow‐up of only 7 days^17^ or less^14^, or no discrimination between the two repair methods among patients with chronic pain^19^. Two studies favoured mesh over suture, as ‘chronic pain in the operated area’ was found only in patients who had a suture repair (4 of 144, 2·8 per cent)^18^, and fewer analgesics were used for patients undergoing mesh repair compared with suture^16^. ‘Pain at rest or during physical activity’ was present more often in the mesh repair group in a single study^5^. Of 38 patients undergoing a mesh repair, six (16 per cent) had pain at rest and 18 (47 per cent) had pain during physical activity, compared with five (4·3 per cent) and 27 (23·5 per cent) respectively of 115 patients who had a suture repair^5^. In another study^3^, there was no significant difference in postoperative pain: 99 of 146 (67·8 per cent) *versus* 97 of 138 (70·3 per cent) in the suture group; after 2 years, 138 (94·5 per cent) and 129 (93·5 per cent) of patients respectively were pain‐free (*P* = 0·450).

Some studies reported additional outcomes to those shown in *Table* 1. One study^5^ reported patients' evaluation of surgery, and another^3^ reported on quality of life, assessed using two scales of health concepts: the Medical Outcomes Study Short Form 36 Health Survey 13 (Medical Outcomes Trust, Waltham, Massachusetts, USA) and the EQ‐5D™‐5 L (EuroQol Group, Rotterdam, the Netherlands). No significant differences were found between the repair groups at any time points.

## Discussion

This systematic review and meta‐analysis comparing the outcomes of elective open mesh and suture repair for umbilical hernia found that mesh repair was associated with a decreased risk of recurrence at the expense of an increased risk of seroma formation. There was no difference between the groups regarding the risks of SSI, haematoma or chronic pain. These findings are in agreement with other reviews^6^, ^23^, in which mesh repair was associated with a lower rate of recurrence and an increased rate of seroma compared with suture.

The present meta‐analysis included both RCTs and observational studies directed at elective repair of umbilical hernia in adults. Although RCTs are considered to deliver the highest level of evidence, it has been suggested^24^ that both RCTs and observational studies conducted on the same question should be included in meta‐analysis, using appropriate methods to adjust for specific biases. In the present systematic review, both RCTs and observational studies were included intentionally, as RCTs demonstrate the efficacy of a treatment in a specific population under controlled circumstances^24^, ^25^, whereas observational studies represent a more diverse group of patients, yielding higher external validity^25^. Although observational studies introduce some heterogeneity, owing to the variations in patient demography and technical elements of repair, it is worth noting that five of the six included RCTs were estimated to have an unclear to high risk of bias, due to poor explanation of the methods applied to adjust for bias. It was therefore considered justifiable to include both RCTs and observational studies, owing to the susceptibility to bias in each study design.

Risk factors affecting the outcome after ventral hernia repair include patient‐ and procedure‐related variables^26^. Patient variables associated with an increased risk of complications after ventral hernia repair include BMI of 30 kg/m^2^ or above, poorly controlled diabetes, smoking, chronic obstructive pulmonary disease, and a history of SSI^27^, ^28^. Procedural variables such as antibiotic prophylaxis, duration of surgery, and use of drains may also affect this risk^26^. Mesh repair may require extended surgical dissection and prolonged operating time, along with risks of local foreign body reaction, seroma formation and contamination^26^. The reporting of patient‐ and procedure‐related variables differed considerably among the included studies, as did assessment of the outcomes SSI, seroma and haematoma. Differences in risks of SSI and seroma might reflect selection bias in the cohort studies, where four reported a significantly higher risk of SSI^1^, ^5^, ^12^, ^20^ and one an increased risk of seroma after mesh compared with suture repair^12^. Surgeons might have chosen mesh over suture repair for patients perceived to have a greater risk of recurrence, increasing the risk of SSI and seroma among the mesh‐repaired patients.

A single RCT^3^ found a significant decrease in the rate of recurrence for small hernia defects; defects of 1–2 cm were associated with a recurrence rate of 2 per cent following mesh repair *versus* 8 per cent after suture closure. In a large cohort study^2^ of both small umbilical and epigastric hernias, significant differences in cumulative recurrence rates were found after mesh *versus* suture repair of 0–1‐cm hernia defects (12 *versus* 21 per cent respectively) and 1–2‐cm defects (8 *versus* 17 per cent). In the present review, specification of hernia size varied too much for a subgroup analysis to be performed, so it was not possible to identify a specific defect size for small hernias for which mesh repair significantly reduced the recurrence rate without increasing the risk of complications.

Recently, the use of internal meshes for pelvic organ prolapse and urinary incontinence has received attention in public media, with an emphasis on the risk of complications such as chronic pain. Court actions against mesh manufacturers have been taken in several countries^29^. Concerns regarding pain with abdominal wall meshes may be less of an issue, but it is worth noting that in the present review the methodology of assessing chronic pain in each study varied markedly and pain results were contradictory, as both mesh and suture repair were favoured in individual studies for the prevention of chronic pain. However, the included large randomized multicentre trial showed that the risk of chronic pain was not altered significantly after mesh *versus* suture repair, when assessed by validated patient‐reported outcome measures (PROMs)^3^. This was confirmed in the large cohort study^2^ for both umbilical and epigastric hernias; this study used a prospective follow‐up questionnaire to assess chronic pain and found no significant difference following mesh or suture repair. Registration and reporting of mesh use should be improved, and the wider use of PROMs, especially those incorporating measurement of recovery after abdominal surgery, advocated^29^, ^30^.

This review has limitations. Considerable heterogeneity was present in the meta‐analyses owing to varying methodologies, and only one RCT^3^ was estimated to have a low risk of bias. Although selection bias in the cohort studies may have favoured suture over mesh to prevent SSI and seroma, preoperative prehabilitation, antibiotic prophylaxis and perioperative factors such as length of incision and surgical technique may have varied considerably between the studies. Lack of standardization regarding postoperative recovery protocols and variations in the duration of follow‐up were further confounders that could not be considered for analysis.

Although elective mesh repair of umbilical hernia imposes an increased risk of seroma, there was still variation in the estimation of this risk. On the basis of lower recurrence rates and no clear evidence of an increased risk of chronic pain, the technique of mesh repair is supported.
